# Common microRNAs Target Established ASD Genes

**DOI:** 10.3389/fneur.2014.00205

**Published:** 2014-10-28

**Authors:** Sharmila Banerjee-Basu, Eric Larsen, Sabina Muend

**Affiliations:** ^1^MindSpec, Inc., McLean, VA, USA

**Keywords:** autism spectrum disorders, microRNA, gene expression regulation, neurodevelopmental disorders, neuropsychiatric disorders, schizophrenia, FMR1

## Complex Genetic Architecture in Autism

Autism spectrum disorder (ASD) encompasses a range of early-onset neurodevelopmental disorders characterized by impaired social interactions and communications, together with repetitive stereotypic behaviors (MIM20895; DSM5). Individuals with ASD can display a broad clinical profile ranging in symptom severity and comorbidities. A strong genetic component underlying ASD has been firmly established; hundreds of genes and chromosomal loci are known to be associated with the disorder and have been cataloged in ASD-specific genetic databases such as AutDB (1). The potential genetic risk factors range from candidate genes reported from genetic association studies to rare, recurrent gene-disruptive mutations identified in ASD individuals using high-throughput technologies such as whole exome sequencing (2). A number of recurrent copy number variants (CNVs) that overlap with related neurodevelopmental and neuropsychiatric disorders such as intellectual disability and schizophrenia have been implicated in ASD as well. Two recent studies, in particular, provide a deeper understanding of the complex genetic architecture of ASD. First, a genome-wide analysis revealed shared risk loci between five major psychiatric disorders including ASD, attention deficit hyperactivity disorder (ADHD), bipolar disorder, major depressive disorder, and schizophrenia (3). The second study examined the detailed structure of the type of genetic liability in ASD and showed that inherited common variants contributed to 49.4% heritability, whereas rare *de novo* variants accounted for only 2.6% (4). Despite this progress in ASD genetics, the underlying perturbations in brain development that contributes to the emergence of specific symptoms along developmental time points, whether in the first years of life in ASD or during adolescence in the case of schizophrenia, remains largely unresolved.

The regulatory mechanisms that orchestrate the precise temporal and spatial patterns of gene expression in the brain are at the forefront of research for brain-based disorders. The importance of various types of non-coding RNAs is being increasingly recognized in this regulatory process, with microRNAs (miRNAs) emerging as a leading candidate. miRNAs are highly conserved small non-coding RNAs approximately 22 nucleotides in length that regulate gene expression mostly by binding to the 3′ UTR of target messenger RNAs (mRNAs). miRNAs recognize their targets primarily through complementarity with the seed sequence at nucleotides 2–8 of the 5′ end of the miRNA. A distinguishing feature of miRNAs lies in the ability of a single miRNA molecule to bind to the recognition site on many mRNAs and subsequently regulate their expression (5). By this mechanism, miRNA–mRNA interactions can potentially modulate expression of hundreds of target genes and influence the corresponding cellular networks. Any disturbances of such a system within a neuronal context could lead to altered brain circuits and synaptic function – processes implicated in disorders such as autism or schizophrenia.

## Role of miRNAs in Psychiatric Disorders

Multiple lines of evidence implicate involvement of miRNAs in schizophrenia. The initial studies involved analyzing differential miRNA expression in individuals with schizophrenia as well as post-mortem brains. However, direct evidence for a contributory role of miRNAs comes from genetic studies. First, the 22q11.2 chromosomal locus, a region in which deletions are strongly associated with schizophrenia risk, contains the gene DGCR8 (DGCR8 microprocessor complex subunit), one of the components of the nuclear miRNA processing complex. In a 22q11.2 deletion mouse model, haploinsufficiency of DGCR8 resulted in the down-regulation of a specific subset of mature miRNAs involved in regulation of synaptic plasticity (6). Moreover, a recent investigation of patients with 22q11.2 deletion syndrome found reduced DGCR8 expression and dysregulated miRNA expression in peripheral blood leukocytes compared to controls (7). Duplications within the 22q11.2 locus containing DGCR8 have been identified as a risk factor for ASD. The localization of DGCR8 within an ASD susceptibility loci and its recently identified regulation by methyl CpG binding protein 2, the protein encoded by the ASD-associated gene MECP2, strongly suggests that a possible link between the role of this gene in miRNA processing and ASD pathogenesis (8). Perhaps the strongest evidence for a contributory role of miRNAs in schizophrenia come from a large GWAS study, where a SNP within an intron of the primary transcript of miR-137 located at chromosome 1p21.3 exceeded the threshold for genome-wide significance (*P* = 1.6 × 10^−11^) (9). Interestingly, miR-137, which is transcribed from miR-137, was recently shown to directly interact with the RAR-related orphan receptor A (RORA) gene, an ASD candidate gene, and potentially interact with a number of additional ASD-associated genes (10).

## Evidence of miRNA Dysregulation in Autism

A number of studies have focused on alteration of miRNA expression patterns in individuals with ASD in recent years. Expression profiling studies in post-mortem brain of individuals with ASD have implicated a number of miRNAs with roles in neurogenesis and plasticity. In a case–control study, Abu-Elneel et al. screened the expression of 466 human miRNAs in the post-mortem cerebellar cortex of 13 individuals with ASDs and an equal number of controls. A total of 28 miRNAs out of 277 were differentially expressed in at least 1 of the autism samples compared to the non-autism controls (11). However, no specific miRNA was uniformly dysregulated across this post-mortem sample set. Using lymphoblastoid cell line (LCL) samples, Talebizadeh et al. evaluated miRNA profiles in 6 individuals with autism compared to matched controls and found altered expression in 9 of the 470 miRNAs in autism samples (12). In an independent study using LCL samples from ASD individuals, 43 miRNAs were found to be differentially expressed in ASD individuals relative to controls (13). Network analysis of target genes from dysregulated miRNAs predicted important pathways involved in nervous system development and plasticity. A recent study using serum samples from 55 individuals with ASD and 55 age- and sex-matched control subjects identified 13 miRNAs that were differentially expressed in ASD individuals compared to the controls (14). However, a consistent view of miRNA dysregulation in ASD is yet to emerge, as only a few overlapping differential miRNA profiles have been identified among these studies.

## Syndromic ASD Genes as Regulators of miRNA Biogenesis

Autism develops as a co-morbid condition in many established single-gene disorders such as fragile X syndrome (MIM 300624), Rett syndrome (MIM 312750), and tuberous sclerosis 1 and 2 (MIM 191100 and MIM 613254), which are caused by mutations in the fragile X mental retardation 1 (FMR1) gene, the methyl CpG binding protein 2 (MECP2) gene, and the tuberous sclerosis 1 (TSC1) and tuberous sclerosis 2 (TSC2) genes, respectively. Interestingly, two genes associated with syndromic ASD, FMR1, and MECP2, have direct links to miRNA biogenesis. The FMR1 encoded protein, FMRP, is a well known sequence-specific RNA binding protein and also interacts with the components of the miRNA processing complex with a likely role at the miRNA maturation step (15, 16). While MECP2 is known as a transcriptional repressor, a recent report by Cheng et al. showed that MECP2 suppresses gene expression at the post-transcriptional level by directly interfering with the assembly of the miRNA processing complex (8). Both FMR1 and MECP2 are intensely studied by the ASD research community regarding their involvement in biochemical pathways, synaptic function, and circuits related to ASD. Importantly, 25% of the affected individuals also develop ASD. Loss of function mutations in MECP2 is the primary cause of Rett syndrome with features of ASD; however, duplications of MECP2-containing loci have been linked to ASD too (17).

## Regulation of MET by miR-34 Family of miRNAs

Although implicated in numerous human cancers, MET receptor tyrosine kinase has emerged as a strong risk gene in ASD. A risk allele located in the promoter region of MET that negatively regulates its transcription has been associated with ASD in independent population cohorts (AutDB, Human Gene module; http://autism.mindspec.org/GeneDetail/MET#HG), providing a functional context for its involvement in this disorder. In a recent paper, Liu et al. showed down-regulation of a set of protein-coding mRNAs including MET in the hippocampus of a fragile X mouse model (*Fmr1 KO*) with a concomitant up-regulation of several miRNAs. Furthermore, the authors showed that three of the elevated miRNAs (miR-34b, miR-340, and miR-148) could down-regulate a reporter construct containing the MET 3’ UTR (18).

**Figure 1:**
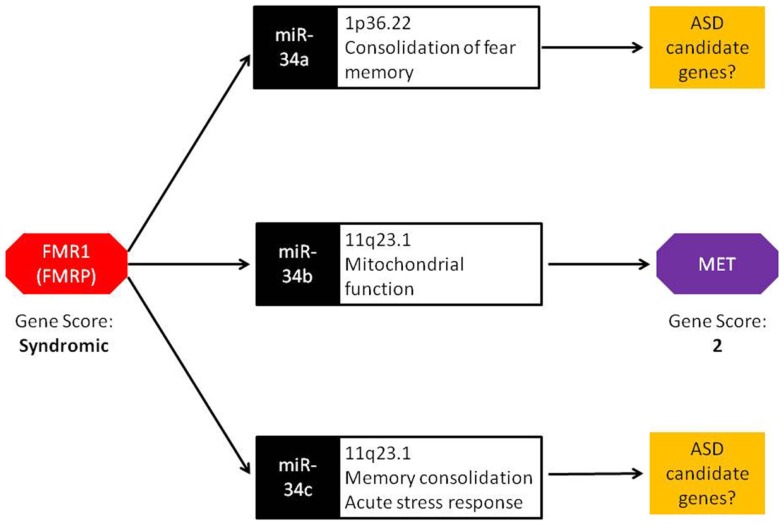
**Bridging the gap: a central role for miRNA in FMR1 and MET crosstalk**. FMRP, the protein encoded by the FMR1 gene, binds to miRNA processing complexes. In FMR1 knockout mice, miRNA expression is significantly up-regulated. In particular, miR-34a, miR-34b, and miR-34c show an absolute fold increase of 41×, 144×, and 83×, respectively. Under normal conditions, the miR-34 family down-regulates MET expression via binding to the 3′ UTR. FMR1 knockout mice display lower MET expression, resulting from the increased ubiquity of miRNAs. Both FMR1 and MET have been scored in the SFARI Gene Scoring module (https://gene.sfari.org/autdb/GS_Home.do). FMR1 is classified as a syndromic ASD gene due to the prevalence of ASD in individuals with fragile X syndrome. MET is classified as a category 2 gene (strong ASD candidate gene) based on replication of genetic association with ASD in multiple independent population cohorts.

The miR-34 family of miRNAs is of particular interest with regards to ASD. While this family has long been recognized in the regulation of various cancer-related cellular processes, recent studies have reported an important role of miR-34 in neuronal development and neuronal disorders. In mouse ES cells, miR-34a functions in the regulation of neurite outgrowth and spinal morphology, indicating its involvement in fundamental neuronal functions (19). Evidence for a role of miR-34 in psychiatric disorders comes from a case–control study of schizophrenia where miR-34a was differentially expressed in blood samples from schizophrenia cases (20). Involvement of other members of miR-34 family in ASD-relevant phenotypes has also been reported. Induction of miR-34c in the central amygdala was observed following acute restraint stress in rodents (21). Additionally, increased expression of miR-34c within the central amygdala led to the onset of anxiety behavior in the same model. In an independent study, Zovoilis et al. showed that miR-34c functions as a negative constraint of memory consolidation in mouse models (22). In a recent study, Dias et al. reported a role for miR-34a within the basolateral amygdala (BLA) in fear memory consolidation (23). Finally, miR-34b and miR-34c were dysregulated in post-mortem brain of individuals with early-stage Parkinson disease and shown to play a role in mitochondrial function and dynamics and oxidative stress (24). Together, miR-34 family members are important regulators of neuronal development, plasticity, and disease. It is worth noting that, in addition to miR-34b, miR-34a, and miR34-c are also up-regulated in *Fmr1 KO* mice (18). These members of the miR-34 family may influence the transcription of other ASD candidate genes similar to miR-34b regulation of MET (Figure 1).

## Conclusion and Future Directions

Despite compelling evidence for the involvement of miRNAs during normal brain development and neuronal disorders, their contribution to ASD pathogenesis is inadequately assessed to date. A systematic identification and characterization of miRNAs targeting high-confidence ASD genes is likely to shed new light into the mechanisms underlying ASD – an important step for developing better treatments for this disorder with increasing prevalence.

## Conflict of Interest Statement

The authors declare that the research was conducted in the absence of any commercial or financial relationships that could be construed as a potential conflict of interest.
